# Bio‐ and paleoreconstructions: Correlates and proxies

**DOI:** 10.1002/ece3.7883

**Published:** 2021-07-13

**Authors:** Marcus Clauss

**Affiliations:** ^1^ Clinic for Zoo Animals, Exotic Pets and Wildlife Vetsuisse Faculty University of Zurich Zurich Switzerland

## Abstract

In paleontology and biology, measures that correlate with specific traits are often used as proxies for these traits without a clear concept of how their discriminatory power is assessed. This note warns about this practice.

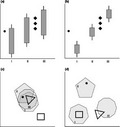

It is common practice to record data patterns (e.g., isotope or dental microtexture signatures, or landmark‐based morphological complexes) in extant animals of known ecology (e.g., trophic categories), take similar measurements in other specimens—whether extant or fossil—and derive interpretations about the corresponding category of the latter based on how their measurements plot onto the “background pattern” derived from the former (Figure 1a–d). This process is often termed “proxy” or “toolkit” development and application.

**FIGURE 1:**
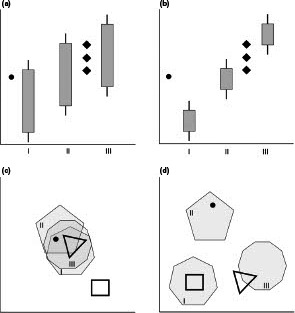
Hypothetical scenarios representing data patterns of “proxy systems” based on a single measurement (a, b) or on a larger number of measurements summarized in the principal component analysis (PCA) or linear discriminant analysis (LDA) plots (c, d) differentiating between categories I‐III. Although all hypothetical patterns show statistically significant correlations or differences between the groups, scenarios a and c have little discriminatory power, in contrast to scenarios b and d. Applications of such proxy systems for deriving the category of additional specimens (symbolized by dots for *n* = 1 specimens, and diamonds, triangle, or square for *n* = 3–4 measurements) should contain, in the method descriptions, clear a priori procedures how results will be interpreted (including results outside of the space covered by the baseline dataset)

Works reporting on these approaches typically contain detailed and stringent descriptions of the sophisticated methods used for data measurement. Sometimes, they contain less stringent methodological descriptions of the derivation of the categories of interest, which might be, for example, taken uncritically from published catalogues (Schradin, 2017). There might also be only limited discussion of why one should assume that the categories used comprise the options theoretically available to the “target” specimens. However, what is often lacking from these works altogether is a stringent methodological description of how the comparison of the data from “target” specimens with the “background pattern” is performed. In other words, extreme methodological rigor in the taking of quantitative measurements and the derivation of quantitative “background patterns” contrasts with little rigor in data comparison.

Two ways of performing these comparisons come to mind:

1. Statistical, quantitative comparisons, where tests are used to state the probability that one or several categories are ascribed to the data of a target species. Such tests evidently do not perform well on *n* = 1 sample sets, as in exceptional fossils.
2. Verbal descriptions of the visual pattern, which either first describe all range overlaps equally and subsequently judge this against previous knowledge of the target species, or use the previous knowledge of the target species already as a starting hypothesis and simply report whether this hypothesis is contradicted by the visual pattern of the proxy. Notably, this approach relies heavily on already existing interpretations and is therefore vulnerable to some degree of circularity, while often rhetorically promoting itself as superior due to the methodological rigor of measurement.

If quantitative data evaluation is considered a hallmark of scientific work, approach (2) appears evidently inferior to, and less rigorous than, approach (1). However, approach (2) additionally loses scientific rigor if, in the methods section, no clear a priori concept is given of how the visual pattern will be interpreted, based on the actual “background pattern” used. For example, a result like the single black dot (for *n* = 1 sample) in Figure 1b and d could be easily ascribed to a category of the background dataset, only by verbal description of the visual assessment (though without any statistical power). However, how would the same data be interpreted against the background patterns depicted in Figure 1a and c? Without describing the process of interpretation a priori in the methods, any given interpretation becomes ad hoc and would have to be labeled as such. In particular, by remaining silent about interpretation criteria in the methods section, approach (2) may appear more rigorous to readers that are biased in terms of the result they expect (as in: “a Diplodocid should group with the ‘herbivores’”) than it actually is.

In approach (2), a hallmark of stringency is whether the interpretation of “intermediate categories” is allowed. If this were outlined in the methods section, as in “a pattern resembling the three diamonds in Figure 1b will be interpreted as an intermediate category between categories II and III,” it would become evident that this requires niche space between these categories. If, for example, category III represented herbivores defined as all animals for which plant material represents ≥90% of the natural diet, and category II, omnivores as those animals for which plant material is less than 90% (but more than 10%), there would obviously be no space between these categories, and hence, allowing an intermediate category (rather than saying that the species can fall in both categories) would require specific justification.

A comparison is instructive with a field where diagnoses based on proxies against a “background pattern” are vital: human medicine. A classic statement in human medicine is that “a correlate does not a surrogate make” (DuBroff, 2017; Fleming & DeMets, 1996), meaning that even though there may be statistical correlations between categories (such as the patterns depicted in Figure 1a and c), this does not automatically mean that the measurements also represent powerful diagnostic proxies. This has been shown repeatedly in biological datasets. Fraser and Theodor (2011) reported highly significant differences in craniodental parameters between ruminant feeding types yet very limited discriminatory power when these were used individually. Allen et al. (2015) found significant differences in various dental measures between three New World monkey feeding types, but variable discriminatory power. The test of whether a system is a powerful proxy does not depend on whether one can demonstrate significant differences between the targeted categories—this is only a necessary but not a sufficient criterion. In this respect, morphophysiological, ecomorphological, or ecophysiological insights emerge much more easily—by the detection of such correlations—than proxy systems of diagnostic power. The latter additionally require discriminatory power that cannot be tested by just assessing differences or correlations. To use an example topic, I was repeatedly involved in the soft‐tissue morphophysiological adaptations of ruminants to browse or grass diets: Although there are general trends that can be demonstrated in statistical terms, there are notable outliers to the overall patterns (e.g., Hertaeg et al., 2021). These do not necessarily invalidate the prevailing morphophysiological relationships and the corresponding functional interpretations, but they indicate that the measures are unlikely to be reliable proxies for feeding types.

Considering that publications are also a means by which our own concept of science is passed on to future generations, I believe it is important to request clear descriptions not only for the methods of data acquisition and tests for correlations but also for the methods of data comparison in the process of extrapolation. Statistical proof of the relevant discriminatory power and not just significant differences between, or correlations with, categories must be provided, for example, by the use of training and test datasets, before claims are accepted that a proxy system has diagnostic power. These tests should be based on realistic scenarios that resemble the intended applications. For example, “leave‐one‐out” procedures should also be applied to whole species and whole taxa (whenever the “target” individuals foreseen for proxy application will represent different species or taxa), not only to a single individual of a species or a single species of a taxon (with the other individuals or species still representing the species’ or taxons’ dataspace in the dataset) (Fulwood et al., 2021). In the case of overlapping dataspaces, the proportion of correct classifications should be supplemented by an indicator of the number of other dataspaces the test case overlapped with. Ultimately, a simple, intuitive assessment of a proxy's or toolkit's quality could be whether one would feel comfortable if decisions in human medicine would be based on its accuracy.

## CONFLICT OF INTEREST

None declared.

## AUTHOR CONTRIBUTION

**Marcus Clauss:** Conceptualization (lead); Writing‐original draft (lead); Writing‐review & editing (lead).

## Data Availability

No data are linked to this manuscript.
